# A longitudinal study comparing the impact of pesticide exposure on cognitive abilities of Latinx children from rural farmworker and urban non-farmworker families

**DOI:** 10.1016/j.ntt.2025.107450

**Published:** 2025-04-13

**Authors:** Monica Soni, Haiying Chen, Milton J. Cepeda, Lesley Berenson, Sydney Smith, Kim A. Anderson, Sara A. Quandt, Thomas A. Arcury, Paul J. Laurienti, Jonathan H. Burdette

**Affiliations:** a Department of Radiology, Wake Forest University School of Medicine, Winston-Salem, NC 27157, USA; b Department of Biostatistics and Data Sciences, Division of Public Health Sciences, Wake Forest University School of Medicine, Winston-Salem, NC 27157, USA; c Department of Psychological Services, Winston Salem Forsyth County Schools, Winston Salem, NC 27105, USA; d Department of Psychiatry, Wake Forest Baptist Health, Winston-Salem, NC 27157, USA; e Department of Environmental and Molecular Toxicology, Oregon State University, 2750 SW Campus Way, Corvallis, OR 97331, USA; f Department of Epidemiology and Prevention, Division of Public Health Sciences, and Center for Worker Health, Wake Forest University School of Medicine, Winston-Salem, NC 27157, USA; g Department of Family and Community Medicine and Center for Worker Health, Wake Forest University School of Medicine, Winston-Salem, NC 27157, USA

**Keywords:** Organophosphates, Organochlorine pesticide, Cognition, Children, WISC-V

## Abstract

Growing evidence shows that pesticide exposure contributes to numerous adverse health effects. Pesticide exposure can be especially problematic for vulnerable populations, and even more so for children in vulnerable populations who are still developing, such as Latinx children. Several studies have demonstrated the negative cognitive effects of prenatal exposure to pesticides, particularly organophosphates (OPs). We previously reported the results from a baseline study (Dobbins et al., 2022) designed to compare the cognitive abilities of 8-year-old children from rural, farmworking families and urban, non-farmworking families. We found that the children from both populations have considerable pesticide exposure, but to different chemicals. The children of farmworkers had greater exposure to OPs, while the children of non-farmworkers had greater exposure to organochlorines (OCs) and pyrethroids. Using the Weschler Intelligence Scale for Children–Fifth Edition (WISC-V), baseline analyses determined that children of non-farmworkers exhibited lower cognitive scores, specifically on the VSI (visual spatial) and VCI (verbal comprehension) indices. The current study examined the longitudinal significance of these findings in the same participants over a 2–3-year period. We present evidence that children from non-farmworking families exhibited significant declines on the FRI (fluid reasoning index) of the WISC-V. The children from farmworker families did not decline, and this longitudinal difference between the groups was significant. Our findings further suggest that these declines in FRI scores are likely due to greater cumulative OC exposure over the entire longitudinal period.

## 1. Introduction

Throughout the past century, pesticide use has massively increased. In the United States (US) alone, over 1 billion pounds of pesticides are used each year (Alavanja, 2009), much of it in the agricultural industry. As such, human exposure to pesticides is unavoidable and is an ongoing cause for concern (Umetsu and Shirai, 2020). In particular, children are especially vulnerable to the toxic effects of pesticides due to their developing bodies and nervous systems (Hyland and Laribi, 2017). The most frequently detected pesticides in rural and urban Latinx children are organochlorines (OCs), organophosphates (OPs), and pyrethroids (Arcury et al., 2023). Though banned in western countries since the 1970s, OCs, the most common being dichlorodiphenyltrichloroethane (DDT), are still omnipresent as they tend to persist in the environment, and they have long-range atmospheric transport potential (Ali et al., 2014). OPs, on the other hand, have extensive use in US agriculture, and some OPs are hydrolyzed to produce dialkylphosphates (DAPs) (Forsberg et al., 2011), which can be found on produce sprayed with OPs (Zhang et al., 2008). Pyrethroids are used widely as insecticides both in homes and commercially in the US (Bradberry et al., 2012).

Children in rural farmworker families (FW) are particularly at risk for exposure to any potential neurotoxic effects of agricultural pesticides such as OPs, since their families live and work in rural agricultural communities, putting them at greater risk of exposure to OPs compared to children from families that live and work in urban communities (Curwin et al., 2005; Dobbins et al., 2022; Hyland and Laribi, 2017; Quandt and Arnold, 2020). In our longitudinal study, we found that when controlling for season, urban children were exposed to greater concentrations of OCs, whereas, rural children were exposed to greater concentrations of pyrethroids and chlorpyrifos (Arcury et al., 2023). Chlorpyrifos is a specific OP that is associated with decreased cognitive abilities (Bouchard et al., 2011; Engel et al., 2011; V. Rauh et al., 2011) and altered brain development (V. A. Rauh et al., 2012) in children with prenatal exposure. In addition, pesticide concentrations in both rural and urban environments are lower in winter and spring seasons compared to summer and fall (Arcury et al., 2023).

There is considerable evidence in both animals and humans that prenatal exposure to OPs impairs fetal growth and development (Peiris-John and Wickremasinghe, 2008). Exposure to OPs, particularly during prenatal development, can result in difficulties with motor coordination, attention, and respiratory issues (Costa, 2006; Naughton and Terry Jr., 2018; Richardson et al., 2019; Roldan-Tapia et al., 2006). It can be argued that the most adverse effect of prenatal OP exposure identified in children is reduced intelligent quotient (IQ) scores (Dobbins et al., 2022). Interestingly, the findings regarding the postnatal effects of OP exposure are inconsistent (Bouchard et al., 2011; Cartier et al., 2015; Dobbins et al., 2022; Marks et al., 2010).

Most studies on OP exposure focus on children in FW families since agricultural exposure is the most common source. Nevertheless, children from non-farmworking families (NFW) living in urban environments are also at risk of exposure to various pesticides. We previously reported that these children are exposed to different classes of pesticides, including persistent OCs and pyrethroids (Arcury et al., 2021). In addition, longitudinal findings determined that these same children are most exposed to OCs (Arcury et al., 2023). We recently published results from a study that used the Wechsler Intelligence Scale for Children fifth edition (WISC-V) to compare IQ scores in Latinx children from rural FW and urban NFW families (Dobbins et al., 2022). That study, Preventing Agricultural Chemical Exposure (PACE5), used baseline IQ scores and pesticide exposure measures from children when they were 8 years old. It was found that the children from NFW families had lower scores on the verbal comprehension index (VCI) of the WISC assessment, and marginally lower scores on the visual spatial index (VSI), compared to those from FW families. When examining the association these differences had with pesticide exposure, it was found that OC pesticide exposure was most strongly associated with these two indices of the WISC-V. Neither measure of OP nor pyrethroid exposure meaningfully affected the observed group differences.

The current study extends the PACE5 baseline findings by examining the 2–3-year longitudinal changes in WISC-V scores in the same children from rural and urban areas. Examinations of the associations with longitudinal pesticide exposure also determined if longitudinal group differences were associated with cumulative exposure to OCs, OPs, or pyrethroids. A lack of baseline differences in any index does not preclude group differences in longitudinal changes, as many of the indices exhibit peak rates of developmental change at different ages.

## 2. Methods

The Preventing Agricultural Chemical Exposure (PACE5) study was designed to identify and compare the effects of overall pesticide exposure on cognitive and brain development in Latinx children from rural FW and urban NFW families. PACE5 was a longitudinal community-based participatory research (CBPR) project performed as a partnership between Wake Forest University School of Medicine and North Carolina Farmworkers Project (Benson, NC; http://ncfwp.org). The current paper expands on the baseline testing that was done in a previous study (Dobbins et al., 2022), and reports the longitudinal findings obtained from follow up visits with the same participants. Since the same participants were used as in the previous study, many of the details below are the same as cited in (Dobbins et al., 2022). The methodology that is the same as the previous study is briefly summarized below, but please refer to (Dobbins et al., 2022) for further details. Any methodology that was specific to this longitudinal study is described in depth below. The Wake Forest University School of Medicine Institutional Review Board approved all PACE5 protocols and procedures, and the study received a Certificate of Confidentiality from the National Institutes of Health.

### 2.1. Participant recruitment

This study recruited children from FW families located in eastern North Carolina and children from NFW families from central North Carolina. A FW family is defined as a participant’s household with at least one adult who has worked on non-organic farms for the past three years. NFW families could not have any adults living in the home that had routine exposure to pesticides through employment or had lived adjacent to agricultural fields within the previous three years. Inclusion criteria for the children included: being 8 years old and having completed the first grade in the United States at the time of entry, self-identified as Latinx, and household incomes below 200 % of the United States federal poverty line. Exclusion criteria included: a life-threatening illness, history of a neurological, physical, or developmental disorder that would interfere with completing the cognitive testing or a brain scan, which was part of the larger study. Please refer to (Dobbins et al., 2022) for more details.

### 2.2. Demographic and life history data collection and measures

Mothers of the participants completed baseline questionnaires that gathered information about the child participant, their family, and their home environment. We continued to collect this same information at each follow up visit. We accounted for the same potential covariates as detailed in (Dobbins et al., 2022), which were child sex, whether the child had pre-k education experiences, season of WISC-V assessment, highest maternal education completed in years, maternal depression using the Center for Epidemiologic Studies Depression Scale (CES–D) (Radloff, 1977), prenatal alcohol and tobacco use, presence of a father in the home, number of siblings, housing conditions (apartment, trailer, or detached home), water sources, and exposure to adverse life experiences using the Adverse Childhood Experiences (ACEs) scale (Felitti et al., 2019).

### 2.3. Study timing and COVID

The study was originally designed to conduct quarterly visits with participants over a 2-year period, with cognitive assessments being held at the beginning and end of the 2 years. However, due to the COVID-19 pandemic, the study was delayed, and necessary adjustments were made. Family recruitment for the study was continuous, beginning in March 2018 and ending in December 2019, with the final participant completing their baseline WISC test in January 2020. Due to COVID, we were unable to continue with our regularly scheduled visits beginning in March 2020. All communication with participants started taking place via phone calls. We dropped off study materials to participants, including pesticide detection wristbands, by leaving them on the porch of homes and picked them up via this same method. This pesticide exposure monitoring process is described in further detail below. Final testing for participants was intended to take place from March 2020 through December 2021. However, final testing was delayed until April 2021 and went through February 2022. Since many participants’ final testing was delayed, the study ended up taking place over more than the planned timeframe of 2 years, with the timeframe for a participant ranging between 2 and 3 years. Some participants had no delay in timing, and those who did have a delay are all included and accounted for in the data below.

### 2.4. Cognitive assessment

Children were administered the Wechsler Intelligence Scale for Children, Fifth Edition (WISC-V, pearsonassessments.com) to assess cognitive abilities. The WISC-V is the standard instrument for the neurobehavioral assessment of a child’s cognitive abilities. Specific cognitive domains are quantitatively assessed with the following five indices: Verbal Comprehension Index (VCI), Visual-Spatial Index (VSI), Fluid Reasoning Index (FRI), Working Memory Index (WMI), and the Processing Speed Index (PSI). The WISC-V also provides a combined index score representing general intellectual ability, termed Full-Scale Intellectual Quotient (FSIQ). Parents were not in the room during the testing procedures to ensure that testing was not interrupted and in keeping with clinical testing and administration standards.

Children were tested in their more proficient language (English or Spanish) by using a language/school report as described in (Dobbins et al., 2022). Two clinical psychologists (one bilingual and one English-speaking) and one bilingual specialist in school psychology completed all assessments. The majority of children were tested in their proficient language by the bilingual testers. The English-speaking psychologist performed six (6) baseline tests in children who were most proficient in speaking English. Whenever possible follow-up testing was performed by the same person that completed the baseline testing. Due to scheduling complexities, this was not always possible. We had 2 primary testers who did the Pre and Post testing. However, 4 of the participants had Pre-tests performed by an additional tester. All of the test administrators were clinically licensed and certified to administer the Pearson WISC-V (level C verification). They also used this test as part of their clinical practice. Pearson has established administration procedures that are highly standardized so that administration by trained and licensed professionals will help ensure reliability of the results.

The test administration took place using the Q-interactive format. Q-interactive is a 1:1 iPad-based testing system that helps administer, score, and report the results of the WISC–V. Test administration on Q-interactive takes place via two iPads connected via Bluetooth. This allows the tester to explain instructions, record and score responses, take notes, and control visual stimuli while the child views and responds to the prompts and tasks provided. The child and tester were seated on opposite sides of a table in a distraction-free, quiet environment during testing. It should be noted that the baseline testing used the fully automated WISC-V version on the iPad. However, on July 14th of 2020, Pearson Publishing removed two subtests (coding and symbol search, both subtests part of the Processing Speed Index - PSI) from the iPad. The administration of the coding and symbol search changed from a fully digital task to a manual task that continued to involve speed, accuracy, and added fine motor dexterity and the ability to manipulate a pencil. Thus, caution should be used when comparing results from this study with studies that used the WISC-V version with the manual subtest administration. Pearson provided a mechanism to rescore the tests in order to be able to compare scores pre- and post- the change in the administration of the test by removing the PSI. We performed this rescoring procedure for all of our data in order to be able to directly compare our baseline and follow-up data. However, caution is still advised when comparing this data to previous literature because scores in the literature may not be adjusted in the same way our scores were adjusted.

In follow-up visits, it became clear that two NFW children had errors in their date of birth entered into the WISC-V software. We were unable to go back and rescore these tests with the correct birth date due to the change in the testing/scoring procedure as described above. However, the age differential due to the error was within a year for both participants, and the clinical psychologist on the study was confident that these differences would not affect the overall scoring in a meaningful way.

### 2.5. Pesticide exposure monitoring

In order to monitor pesticide exposure in the participants, we utilized silicone wristbands which were able to detect the exposure to pesticides in the environment. 72 different pesticides were assessed, with the most frequent classes being OCs, OPs, and pyrethroids (Arcury et al., 2023). At the end of the interviews, interviewers gave participants the wristbands and provided instructions on how they should be worn (Anderson et al., 2017). The participants wore the wristbands for a seven-day period and after the seven-day period, the wristbands were removed from the child and placed in a Teflon bag. The wristbands remained in the Teflon bag until retrieved by interviewers and they were then shipped to the laboratory for analysis. Please refer to (Dobbins et al., 2022) for further details.

Study design dictated that wristbands should be collected quarterly for pesticide exposure. However, some participants had missing wristbands, mostly due to COVID with some others being due to factors such as lost or torn storage bags. During COVID, we stopped in-person study visits but continued to collect wristbands from participants who had not reached their scheduled 2-year completion date. When a participant reached their planned 2-year final visit, we stopped giving them quarterly wristbands due to costs that were not budgeted. When in-person visits were re-started, participants that missed their planned 2-year visit were scheduled for a final visit as soon as possible. Thus, wristbands were not collected for quarters that passed from the planned 2-year visit to the actual final visit. All participants were given a wristband to wear before their final study visit.

For all instances of missing data, the number of different pesticides detected for each class of pesticide were imputed within subject using the following methodology. First, all collected data were assigned to a quarter of the year. Because there are many scheduling complexities involved in a CBPR project such as this, precise quarterly dates of wristband deployment were not always possible. In the event of two collection dates occurring within a quarter (for example, one wristband collected at the beginning of January and the other at the end of March), the wristbands were assigned to the quarter that most closely aligned with dates from other study years in that participant. In the above example, if wristbands were collected in April for the other study years, the March wristband would be assigned to the 2nd quarter. On the other hand, if wrist bands were consistently collected in December, the January wristband would be assigned to the 4th quarter. Data was deemed missing if there were quarters with no assigned wristbands after this alignment procedure. For quarters with missing wristbands, the data were imputed by averaging the other wristbands assigned to the same quarter within subject. Most missing quarters had 2 other matching quarters to be used for imputation. Some subjects only had one other wristband with the same quarter assignment. For these imputations, the value from the existing quarter was used for the missing quarter. Across the total of 1280 wristband samples used in this study, 258 were imputed with 72 imputed from a single matching quarter. All participants had at least one wristband from each of the four quarters of the year. The final exposure values that were used for follow-up included the total cumulative exposure over the duration that participant was in the study. Our hypothesis was that the adverse effects of exposure accumulate in a nonlinear manner, so we also included baseline values. Ideally, one would have a measure of total lifetime exposure, but such a measure was not available in the current study.

### 2.6. Data analysis

Descriptive statistics for participant and household characteristics and pesticide exposures are presented by rural status at baseline and follow-up. Bivariate analyses were used to assess the differences in participant demographics and pesticide exposures by rural status using Chi-square tests, Fisher’s Exact, Kruskal-Wallis tests, or *t*-tests as appropriate. Participant and household characteristics were tested with a significance level of <0.1 and were included as covariates in statistical models. Presence of a learning disability was not included as a covariate because it may be a causal intermediary for the effects of pesticides on cognitive function (Ananth and Schisterman, 2017). Current diagnosis of ADHD was recorded based on maternal responses and could be associated with the cognitive assessment scores; however, it was not included as a covariate due to its low frequency reported at baseline (1 NFW child and 0 FW children) and follow-up (2 NFW children including the one from baseline and 2 FW children). Baseline marital status was confounded with the presence of father in household and was also excluded as a covariate. Due to disruptions in the schedule because of COVID, we adjusted for time between WISC-V assessments to account for participants having varying lengths of time between baseline and follow-up.

Linear mixed-effects models assessed the WISC-V scores at baseline and follow-up. Models included the rural status of the participant, timepoint of the test (baseline or follow-up), and their interactive effect. The adjusted models also included the covariates identified with *p* < 0.1 in the bivariate analysis. WISC-V assessment measures and participant/household characteristics collected at multiple follow up visits with p < 0.1 were included as time-varying covariates in the adjusted models. Separate analyses were performed adjusting for each of the three most prominent classes of pesticides found within these populations: organochlorines (OCs), organophosphates (OPs), and pyrethroids. In order to make comparisons within each timepoint, linear mixed-effects models that adjusted for time between tests were used to make estimates at baseline and at the average follow-up time. The Tukey-Kramer method was used to adjust for multiple comparisons. *P*-values were considered ed. significant at *p* < 0.05.

## 3. Results

Table 1 reports the demographic information from participants as well as their mothers. We had 125 participants at baseline and 107 participants at follow-up for collecting demographic information. For the WISC-V assessments, we had 123 participants at baseline and 107 at follow-up. The bolded P-values in all of the tables represent values less than 0.05 when comparing characteristics of NFW and FW participants. Several demographic differences were present, such as attending pre-kindergarten (higher percentage in the NFW), maternal education (more years in the NFW), total adults in the household (more in the NFW), testing season at baseline and follow-up, testing language (greater percentage of the NFW tested in English), incidence of learning disabilities (greater in the NFW), and number of adverse experiences (more in the FW).

Table 2 reports the number of different pesticide detections for both groups at baseline and follow-up (the imputed total cumulative number of detections). Table 2 also compares the baseline and follow-up mean (SD), median (IQR), and the minimum and maximum detections found in NFW and FW children for OCs, OPs, and pyrethroids. We previously reported the significant differences in OCs, OPs, and pyrethroids at baseline for NFW versus FW children (Arcury et al., 2021), and a complete analyte list, detection limits, quantitation limits and chromatographic conditions are given in the electronic supplementary material for that manuscript describing in detail the wrist band data. The follow-up numbers are much larger than the baseline numbers because the follow-up numbers represent all of the wrist band data combined, including the baseline data. For each participant, that turned out to be approximately 10 time points; thus, these follow-up numbers are in the range of 10× the baseline numbers. For most analytes, identification and quantitation were made on the DB-17MS column, and the DB-XLB column was used for confirmation. Our longitudinal data show significant differences at follow-up were present for OCs and OPs and not pyrethroids. Like with the baseline data, the follow-up levels of OC detection were greater in the NFW children, and the OPs were greater in the FW children.

Table 3 compares cognitive scores collected at baseline testing and at the follow-up for the different WISC-V indices for the NFW and FW groups. Additionally, the calculated difference between NFW and FW changes from baseline occurred by subtracting both groups’ estimated change from baseline. The bolded *P*-values in the forthcoming tables (Tables 3–7) represent significant values. For the NFW children, the Fluid Reasoning Index (FRI) significantly decreased between baseline and follow-up, and the Full Scale IQ (FSIQ) showed marginal significance. In the FW children, the FSIQ decreased significantly between baseline and follow-up, with marginally significant decrease in Working Memory Index (WMI). Longitudinal significant differences between NFW and FW from baseline to follow-up were seen for the FRI and WMI indices. For FRI, the scores for the NFW significantly decreased whereas the scores for the FW slightly increased. For WMI, the scores for NFW children increased, but the scores for FW children decreased.

Table 4 shows cognitive scores *after adjusting for demographic covariates* that were different between groups. Note that all values in the tables are model estimates, and adding in the covariates to the statistical analyses altered the score estimates. The only finding that remained significant was the difference between NFW and FW change from baseline for the FRI index. After adjustment, both groups exhibited non-significant score decreases, but the NFW scores decreased more than the FW scores. Of note, the difference between NFW and FW change from baseline for the WMI index approached significance after adjusting for covariates.

Adjusted for significant demographic variables shown in Table 1: Maternal Education, Father in home, Pre-Kindergarten experience, Number of adults in household, Number of adverse family experiences, Season of WISC-V administration, language of WISC-V administration, and duration between tests.

The values in Tables 5, 6, and 7 are the model estimates that not only adjust for the same covariates as in Table 4, but also for cumulative exposure to OCs, OPs, and pyrethroids, respectively. The cumulative exposure is the total number of pesticides detected in each pesticide class for the length of the study calculated as the summation from the seasonal wristbands. Table 5 adjusts for OCs and shows that the estimated difference between NFW and FW change from baseline for the FRI index was reduced by nearly half of the value shown in Table 4 (before the OCs adjustment). The estimate changed from − 5.72 in Table 4 to − 2.99 in Table 5, and this change was no longer significant after accounting for OC exposure. When adjusting for OPs (Table 6) and pyrethroids (Table 7), the difference estimates for the FRI index changed minimally. For the OP analysis, the estimate decreased slightly to − 5.19 and was nearly significant. For the pyrethroid analysis, the estimate increased to 6.16 and remained significant. No other cognitive change scores were significant after further adjusting for cumulative pesticide exposure, though WMI showed marginal significance after accounting for OPs and pyrethroids.

## 4. Discussion

Given the prevalence of pesticides in our environment and the known neurodevelopmental changes throughout childhood, it remains important to research the neurocognitive effects of different pesticides on children. Here, we present longitudinal evidence that the effects of pesticide exposure during early childhood differ based on the environment in which the child resides. In our baseline study, we found that children from NFW families exhibited poorer performance most notably on the VCI index of the WISC-V compared to children from FW families (Dobbins et al., 2022). In the current study, we examined the longitudinal validity of these findings and confirmed that children of NFW families exhibit poorer cognitive performance compared to children from FW families. We found that VCI differences reported at baseline did remain on the follow-up. However, there were no VCI changes observed longitudinally. Rather, the present study revealed *longitudinal significance* for the FRI of the WISC-V after adjusting for covariates. Specifically, the FRI showed a significant longitudinal decrease in the children of NFW families when compared to the children of FW families.

The present study replicated general baseline results, but for different measures of the WISC-V. These differences likely reflect that the participants in the present study were 2–3 years older compared to when they were tested for baseline results and were, therefore, developing different cognitive components and skills at this time. The VCI (verbal comprehension) and VSI (visual spatial) indices of the WISC-V are associated with reasoning and concept formation, representing different aspects of similar cognitive traits (Dobbins et al., 2022). The FRI (fluid reasoning index) measures general problem-solving abilities and has been shown to develop most rapidly in children 8–10 years old (McArdle et al., 2002). It has also been found that age differences exist in the dynamics of fluid abilities and achievement, and the effects are stronger during childhood and early adolescence (Ferrer and McArdle, 2004) whereas verbal development tends to take place at a more rapid rate earlier in life (Law et al., 2008). So, it is not surprising that VCI and VSI differences were seen on our baseline data, and FRI differences were present in the longitudinal data. Given the increased OC exposure in NFW and the finding that the FRI of children from NFW families ultimately declined over the 2–3 year study period, this indicates a strong possibility that OCs are negatively impacting the cognitive development of children from NFW families as compared to children from FW families (Arcury et al., 2021).

We readily accept the limitations of this study, as detailed below in the “Potential Limitations” section, due to the change in administration of the WISC-V and Covid complications. However, even with these limitations, the results presented here indicate that the cognitive development of children from NFW families is more negatively impacted by pesticides, likely due to more OC exposure, compared to children from FW families. Our observational study design does not allow causal inference, but our findings do imply a strong association between OC exposure and FRI changes, and further research utilizing mediation analyses should investigate these associations further. Although children from FW families also have exposures to various pesticides, such as OPs, the differences in the pesticides used in rural versus urban environments appear to have different effects on cognitive development, with a more adverse impact on the cognitive development, specifically fluid reasoning, of NFW children. When children are 8–10 years old, as the participants were throughout our study, their FRI is developing most rapidly (McArdle et al., 2002). Therefore, this supports our longitudinal findings presented here that the FRI was the portion of the WISC-V to obtain significant longitudinal differences when compared between Latinx children from rural versus urban populations.

### 4.1. Potential limitations

As discussed above, caution is recommended when comparing results from this study with studies that used the WISC-V version with the manual subtest administration. Pearson provided a mechanism to rescore the tests to be able to compare pre- and post- the change in the administering of the test, taking out any data that was affected by the change. Although we performed this rescore with all of our data in order to be able to directly compare our data at the beginning and end of this study, caution is advised when comparing this data to previous literature. Therefore, a limitation of the present study is that it is difficult to directly compare results to other literature unless they also rescored the WISC-V the same way. More specifically, when we rescored the baseline tests, this led to us having to drop out the processing speed index (PSI) due to the different scoring mechanisms. All of the scores we used in this study have the PSI eliminated, so we have to also be careful when comparing full-scale IQ (FSIQ) to other studies. Due to this rescoring, baseline scores presented here are not the same as the baseline scores presented in (Dobbins et al., 2022).

Additionally, COVID posed a major limitation to our study, as it disrupted our study timeline and caused us to improvise our data collection methods to follow CDC guidelines. We are proud of the extensive efforts of our team to continue this important study during the time of COVID restrictions but readily acknowledge that deficits in the data naturally occurred during that time. In addition, COVID lockdown is thought to be related to declines in educational achievement in children, particularly in low-income families (Andrew et al., 2020; Balayar and Langlais, 2022; Ribeiro et al., 2023). However, this does not explain the differences we observed between FW and NFW children. It is possible, that for unknown reasons, there were greater levels of parental involvement in the FW children as that factor has been associated with better outcomes during the COVID lockdown (Balayar and Langlais, 2022).

In addition to the restricted location of North Carolina, the participants in our study can also be considered a convenience sample, both of which limit generalizability. In addition, our pesticide exposure methods utilizing the wristbands just measured pesticide exposure as opposed to dosage, so we do not know how much of the pesticides the children actually had in their systems. Further research should attempt to measure pesticide dosage and investigate the effects of this on the cognitive development of children from FW and NFW families. Finally, we will re-emphasize that two NFW children had errors in their date of birth, and we were unable to go back and rescore these WISC-V tests with the correct birth date due to the change in the testing/scoring procedure. However, the project’s clinical psychologist reported that these differences should not have affected the overall scoring in a meaningful way.

## 5. Conclusion

This study compared longitudinal change in cognitive performance between Latinx children from FW and NFW communities and found a significant difference for the Fluid Reasoning Index (FRI) of the WISC-V. This difference was driven by significant declines in FRI in the NFW population with no meaningful change in the FW population. It is likely that OC exposure is contributory given the greater proportion of children with detectable exposure in the NFW children and that when accounting for the OC exposure, the difference between the two populations was no longer present. Previously, we have reported baseline differences between the two groups in the VCI and VSI portions of the WISC-V. Thus, the differences in age across the longitudinal results reported here likely reflect differences in age of developing cognitive processes, with fluid reasoning developing later than the visual and verbal processes. Clearly, limiting exposure to the ubiquitous environmental pesticides during all stages of childhood is likely beneficial to cognitive development.

## Figures and Tables

**Table 1:** Population and WISC-V Demographics for Children in Farmworker and Non-Farmworker Families.

		Baseline			Follow-up		
		
		NFW *N* = 53 (%)	FW *N* = 70 (%)	p-value	NFW *N* = 46 (%)	FW *N* = 61 (%)	p-value

Sex			0.9175			0.6763
	Female	27 (50.9)	35 (50.0)		26 (56.5)	32 (52.5)	
	Male	26 (49.1)	35 (50.0)		20 (43.5)	29 (47.5)	
Age (months)		102.8 (98.4, 106.5)	100.0 (98.1, 104.5)	0.1093	136.7 (132.5, 140.2)	134.5 (130.7, 139.4)	0.1164
*Pre-Kindergarten		26 (49.1)	15 (21.4)	**0.0013**	22 (47.8)	14 (23.0)	**0.0070**
*Maternal education (years)				**0.0139**			**0.0057**
	0–6	13 (24.5)	31 (44.3)		10 (21.7)	26 (42.6)	
	6–13	31 (58.5)	36 (51.4)		29 (63.0)	34 (55.7)	
	13 +	9 (17.0)	3 (4.3)		7 (15.2)	1 (1.6)	
Maternal birth country				0.4210			0.3423
	Mexico	39 (73.6)	57 (81.4)		34 (73.9)	51 (83.6)	
	US	5 (9.4)	3 (4.3)		4 (8.7)	2 (3.3)	
	Other	9 (17.0)	10 (14.3)		8 (17.4)	8 (13.1)	
Prenatal Substance Use							
	Tobacco	1 (1.9)	0 (0.0)	0.4309	0 (0.0)	0 (0.0)	N/A
	Alcohol	1 (1.9)	1 (1.4)	1.0000	1 (2.2)	1 (1.6)	1.0000
*Father in home		49 (92.5)	57 (81.4)	0.0794	43 (93.5)	49 (80.3)	0.0524
Number of siblings^1^			1.0000			0.7635
	0	3 (5.9)	4 (5.7)		2 (4.4)	4 (6.6)	
	1	13 (25.5)	17 (24.3)		14 (30.4)	13 (21.3)	
	2	17 (33.3)	23 (32.9)		15 (32.6)	21 (34.4)	
	3+	18 (35.3)	26 (37.1)		15 (32.6)	23 (37.7)	
*Total adults in household				**0.0387**			**0.0473**
	1	3 (5.7)	12 (17.1)		3 (6.5)	11 (18)	
	2	39 (73.6)	52 (74.3)		33 (71.7)	45 (73.8)	
	3+	11 (20.8)	6 (8.6)		10 (21.7)	5 (8.2)	
Married at baseline		49 (92.5)	57 (81.4)	0.0794	43 (93.5)	49 (80.3)	0.0524

WISC-V Assessment Measure							
							
		*n* = *53*	*n* = *70*		*n* = *46*	*n* = *61*	

**Testing Language				0.0503			**0.0440**
	Spanish	13 (24.5)	29 (41.4)		11 (23.9)	26 (42.6)	
	English	40 (75.5)	41 (58.6)		35 (76.1)	35 (57.4)	
**Testing Season				**<0.0001**			**<0.0001**
	DEC-FEB	10 (18.9)	15 (21.4)		13 (28.3)	5 (8.2)	
	MAR-MAY	4 (7.6)	33 (47.1)		3 (6.5)	23 (37.7)	
	JUN-AUG	30 (56.6)	6 (8.6)		25 (54.4)	15 (24.6)	
	SEP-NOV	9 (17.0)	16 (22.9)		5 (10.9)	18 (29.5)	
Repeated Measures		*n* = *53*	*n* = *70*		*n* = *46*	*n* = *61*	
Maternal Depression^2^		6 (12.2)	4 (6.1)	0.3206	4 (8.7)	2 (3.3)	0.3988
**Adverse Experiences				0.4065			**<0.0001**
	0	21 (39.6)	34 (48.6)		26 (56.5)	17 (27.9)	
	1	17 (32.1)	23 (32.9)		11 (23.9)	43 (70.5)	
	2+	15 (28.3)	13 (18.6)		9 (19.6)	1 (1.6)	
Learning Disability		9 (17.0)	2 (2.9)	**0.0093**	11 (23.9)	7 (11.5)	0.0886
ADHD		3 (5.7)	0 (0.0)	0.0774	2 (4.7)	2 (3.3)	1.0000

* Included as covariates in adjusted models for WISC-V scores at baseline and follow-up.

** Included as time-varying covariates in adjusted models for WISC-V scores at baseline and follow-up.

1 n = 123;

2 n = 115;

3 n = 104

**Table 2:** Pesticide exposure detections for Children in Farmworker and Non-Farmworker Families.

	Baseline Exposure			Total Cumulative Exposure		
		
	NFW	FW	p-value	NFW	FW	p-value
						
	*n* = *52*	*n* = *70*		*n* = *44*	*n* = *61*	

Organochloride						
Mean (SD)	3.77 (1.49)	2.36 (1.73)	**<0.0001**	42.11 (11.49)	30.6 (9.14)	**<0.0001**
Median (IQR)	4 (3, 5)	3 (1, 4)	**<0.0001**	44.50 (36.25, 49.75)	30 (24.50, 37.00)	**<0.0001**
Min, Max	0, 6	0, 6		5.5, 64	9.83, 51.5	
Organophosphate						
Mean (SD)	0.52 (0.54)	0.76 (0.58)	**0.0224**	5.55 (3.61)	8.78 (3.02)	**<0.0001**
Median (IQR)	0.5 (0,1)	1 (0, 1)	**0.0250**	6 (2.5, 7.92)	9 (7.5, 11)	**<0.0001**
Min, Max	0, 2	0, 2		0, 13.17	0, 13.5	
Pyrethroid						
Mean (SD)	1.65 (1.30)	1.17 (1.33)	**0.0474**	21.33 (8.53)	23.31 (8.2)	0.2345
Median (IQR)	2 (0,3)	1 (0, 3)	**0.0340**	20.25 (15.5, 27.92)	22 (19, 28.33)	0.1735
Min, Max	0, 4	0, 6		3, 40.5	1, 46.50	

**Table 3:** Longitudinal Changes for WISC-V indices for Children in Farmworker and Non-Farmworker Families.

	Non-Farmworker				Farmworker				Difference between NFW and FW Change from Baseline	
		
	Baseline	Follow-up	Estimated Change from Baseline	Adj P-value	Baseline	Follow-up	Estimated Change from Baseline	Adj P-value		
				
	Mean (SE)	Mean (SE)			Mean (SE)	Mean (SE)			Estimate	P-value

**FSIQ**	86.55 (1.66)	83.28 (1.57)	− 3.28	0.057	88.69 (1.46)	85.78 (1.37)	− 2.91	**0.044**	− 0.37	0.827
**FRI**	92.51 (1.68)	87.73 (1.72)	− 4.78	**0.042**	90.95 (1.46)	91.74 (1.50)	0.79	0.955	− 5.57	**0.020**
**VSI**	90.35 (1.51)	88.04 (1.88)	− 2.31	0.372	91.44 (1.33)	88.46 (1.64)	− 2.98	0.078	0.67	0.725
**VCI**	85.52 (1.94)	83.26 (1.90)	− 2.26	0.499	88.83 (1.71)	89.40 (1.66)	0.58	0.976	− 2.84	0.184
**WMI**	86.15 (1.74)	87.95 (1.66)	1.79	0.695	90.82 (1.53)	87.13 (1.45)	− 3.70	0.050	5.49	**0.013**

P-values are adjusted for multiple tests using Tukey-Kramer; FSIQ = Full Scale IQ, FRI=Fluid Reasoning Index, VSI=Visual Spatial Index, VCI=Verbal Comprehension Index, WMI=Working Memory Index.

**Table 4:** Longitudinal Changes for WISC-V indices, *Adjusted for Demographic Covariates*, for Children in Farmworker and Non-Farmworker Families.

	Non-Farmworker						Farmworker						Difference between NFW and FW Change from Baseline	
		
	Baseline		Follow-up		Estimated Change from Baseline	Adj P-value	Baseline		Follow-up		Estimated Change from Baseline	Adj P-value		
				
	Mean	SE	Mean	SE			Mean	SE	Mean	SE			Estimate	P-value

**FSIQ**	86.09	4.63	85.99	5.23	−0.10	1.000	91.07	4.53	91.75	5.24	0.69	1.000	−0.79	0.672
**FRI**	93.14	5.99	85.42	6.83	−7.72	0.918	91.55	5.90	89.55	6.86	−2.00	0.998	−5.72	**0.029**
**VSI**	94.87	5.25	83.08	6.07	−11.79	0.673	99.28	5.13	87.89	6.15	−11.39	0.697	−0.39	0.848
**VCI**	83.74	5.61	87.78	6.42	4.04	0.982	92.43	5.51	98.40	6.45	5.97	0.946	−1.93	0.386
**WMI**	82.72	5.41	94.34	6.20	11.61	0.699	89.22	5.33	96.54	6.22	7.31	0.902	4.30	0.059

P-values are adjusted for multiple tests using Tukey-Kramer.

**Table 5:** Longitudinal Changes for WISC-V Indices, *Adjusted for Demographic Covariates and Total OC Exposure*, for Children in Farmworker and Non-Farmworker Families.

	Non-Farmworker						Farmworker						Difference between NFW and FW Change from Baseline	
		
	Baseline		Follow-up		Estimated Change from Baseline	Adj P-value	Baseline		Follow-up		Estimated Change from Baseline	Adj P-value		
				
	Mean	SE	Mean	SE			Mean	SE	Mean	SE			Estimate	P-value

**FSIQFRI**	86.40	4.64	86.70	5.22	0.31	1.000	91.04	4.53	90.74	5.25	−0.29	1.000	0.60	0.778
**FRI**	93.32	5.94	86.79	6.73	−6.54	0.945	91.64	5.83	88.10	6.77	−3.55	0.990	−2.99	0.300
**VSI**	95.09	5.29	83.65	6.06	−11.44	0.693	99.54	5.15	86.92	6.21	−12.62	0.628	1.18	0.622
**VCI**	84.83	5.62	88.28	6.39	3.45	0.989	92.42	5.50	96.50	6.46	4.09	0.982	−0.63	0.805
**WMI**	82.40	5.45	93.72	6.21	11.32	0.715	89.18	5.35	97.41	6.26	8.23	0.867	3.09	0.228

P-values are adjusted for multiple tests using Tukey-Kramer.

Adjusted for Maternal Education, Father in home, Pre-Kindergarten experience, Number of adults in household, Number of adverse family experiences, Season of WISC-V administration, language of WISC-V administration, and duration between tests.

**Table 6:** Longitudinal Changes for WISC-V indices, *Adjusted for Demographic Covariates and Total OP Exposure*, for Children in Farmworker and Non-Farmworker Families.

	Non-Farmworker						Farmworker						Difference between NFW and FW Change from Baseline	
		
	Baseline		Follow-up		Estimated Change from Baseline	Adj P-value	Baseline		Follow-up		Estimated Change from Baseline	Adj P-value		
				
	Mean	SE	Mean	SE			Mean	SE	Mean	SE			Estimate	P-value

**FSIQ**	86.66	4.68	86.01	5.22	−0.66	1.000	91.52	4.58	90.90	5.28	−0.62	1.000	−0.04	0.986
**FRI**	93.30	6.08	85.51	6.84	−7.78	0.917	91.82	5.98	89.22	6.93	−2.60	0.997	−5.19	0.064
**VSI**	95.58	5.25	83.21	5.99	−12.37	0.631	100.25	5.12	86.39	6.13	−13.86	0.547	1.49	0.503
**VCI**	85.14	5.62	87.73	6.35	2.59	0.995	93.08	5.51	96.49	6.43	3.41	0.989	−0.82	0.731
**WMI**	82.72	5.49	94.35	6.21	11.63	0.701	89.36	5.40	96.42	6.29	7.06	0.914	4.57	0.062

P-values are adjusted for multiple tests using Tukey-Kramer.

Adjusted for Maternal Education, Father in home, Pre-Kindergarten experience, Number of adults in household, Number of adverse family experiences, Season of WISC-V administration, language of WISC-V administration, and duration between tests.

**Table 7:** Longitudinal Changes for WISC-V indices, Adjusted for Demographic Covariates and Total Pyrethroid Exposure, for Children in Farmworker and Non-Farmworker Families.

	Non-Farmworker						Farmworker						Difference between NFW and FW Change from Baseline	
		
	Baseline		Follow-up		Estimated Change from Baseline	Adj P-value	Baseline		Follow-up		Estimated Change from Baseline	Adj P-value		
				
	Mean	SE	Mean	SE			Mean	SE	Mean	SE			Estimate	P-value

**FSIQ**	86.39	4.65	85.87	5.21	−0.52	1.000	91.17	4.54	91.66	5.23	0.49	1.000	−1.01	0.586
**FRI**	93.54	5.97	84.94	6.73	−8.60	0.888	91.96	5.86	89.51	6.76	−2.44	0.997	−6.16	0.019
**VSI**	94.75	5.30	83.07	6.06	−11.67	0.682	99.38	5.17	88.20	6.16	−11.18	0.711	−0.49	0.812
**VCI**	84.57	5.64	87.59	6.39	3.02	0.992	92.39	5.52	97.67	6.43	5.28	0.962	−2.26	0.314
**WMI**	82.67	5.47	94.27	6.21	11.60	0.703	89.28	5.38	96.63	6.24	7.35	0.902	4.25	0.064

P-values are adjusted for multiple tests using Tukey-Kramer.

Adjusted for Maternal Education, Father in home, Pre-Kindergarten experience, Number of adults in household, Number of adverse family experiences, Season of WISC-V administration, language of WISC-V administration, and duration between tests.

## Data Availability

Data will be made available on request.
